# Infratentorial Pressure Monitoring in Cerebellar Stroke: Feasibility and Prognostic Utility

**DOI:** 10.1007/s12028-025-02391-1

**Published:** 2025-10-09

**Authors:** Sae-Yeon Won, Eva Herrmann, Anne Neumeister, Jonas Hagemeier, Daniel Dubinski, Alhalabi T. Obada, Bedjan Behmanesh, Joshua D. Bernstock, Thomas M. Freiman, Olaf Lademann, Artem Rafaelian, Jens-Christian Schewe, Alexander Storch, Andreas W. Unterberg, Johannes Walter, Matthias Wittstock, Nazife Dinc, Florian Gessler

**Affiliations:** 1 Department of Neurosurgery, Rostock University Medical Center, Rostock, Germany; 2 Department of Medicine, Institute of Biostatistics and Mathematical Modelling, Goethe University, Frankfurt Am Main, Germany; 3 Department of Neurosurgery, Jena University Hospital, Jena, Germany; 4 Department of Neurosurgery, University of Heidelberg, Heidelberg, Germany; 5 Department of Neurosurgery, Brigham and Women’s Hospital, Harvard Medical School, Boston, MA USA; 6 Department of Anesthesiology, Intensive Care Medicine and Pain Therapy, University Medical Centre, Rostock, Germany; 7 Department of Neurology, Rostock University Medical Center, University of Rostock, Rostock, Germany

**Keywords:** Cerebellar stroke, Cerebellar hemorrhage, Supratentorial pressure monitoring, Infratentorial pressure monitoring, Predictive model

## Abstract

**Background:**

Although supratentorial intracranial pressure (ICP) monitoring represents the current standard in neurocritical care, its validity for assessing infratentorial pathologies remains uncertain. This multicenter, prospective study aimd to (1) evaluate the feasibility and clinical utility of infratentorial ICP monitoring in acute posterior fossa pathologies and (2) develop a prognostic model for functional outcomes based on infratentorial pressure dynamics.

**Methods:**

We conducted a prospective cohort study across three tertiary neurovascular centers in Germany (2021–2024), enrolling 35 consecutive patients with cerebellar stroke requiring surgical decompression and external ventricular drainage. All participants underwent simultaneous supratentorial and infratentorial ICP monitoring for seven posteroperative days. Functional outcomes were assessed using the modified Rankin scale at discharge and at six-month follow-up (FU). The primary end point was the comparison of ICP gradients between compartments; secondary analyses evaluated the association between infratentorial ICP and functional outcomes.

**Results:**

The mean infratentorial ICP was significantly higher than the supratentorial ICP (11.9 mm Hg [95% confidence interval (CI) 10.5–13.3] vs. 8.8 mm Hg [95% CI 7.4–10.1], *P* < 0.001). Patients with unfavorable outcomes had significantly higher infratentorial ICP values compared with those with favorable outcomes at FU (13.1 mm Hg [95% CI 11.1–15.1] vs. 9.5 mm Hg [95% CI 6.8–12.1], *P* = 0.042). Multivariate logistic regression analysis identified a novel scoring system—calculated as patient age (years) plus four times the mean infratentorial ICP (mm Hg)—as a significant predictor of unfavorable outcomes at both discharge and FU (*P* < 0.001 and *P* = 0.001, respectively), with area under the curve (AUC) values of 0.88 and 0.89. A cutoff value of 115 was established to predicting unfavorable outcomes at FU.

**Conclusions:**

This study establishes that infratentorial ICP monitoring (1) reveals clinically significant pressure gradients undetctable by supratentorial measurement alone, (2) provides superior prognostic information compared with conventional monitoring, and (3) can be safely implemented with 94% technical success rate. These findings advocate for the integration of infratentorial ICP assessment into the neurocritical care paradigm for posterior fossa pathologies.

**Supplementary Information:**

The online version contains supplementary material available at 10.1007/s12028-025-02391-1.

## Introduction

Intracranial pressure (ICP) monitoring serves as a cornerstone in the neurocritical management of patients with traumatic brain injury, stroke, and other acute neurological disorders [1–5]. Current clinical guidelines for severe traumatic brain injury provide detailed recommendations for ICP monitoring and management of intracranial hypertension yet notably lack anatomical specificity regarding lesion location [1]. Although supratentorial ICP measurement remains the standard of care in most institutions, the clinical relevance of infratentorial ICP assessment remains poorly characterized despite the distinct pathophysiological considerations in posterior fossa pathology.

Emerging evidence challenges the presumption of uniform ICP dynamics. A recent a systematic review and meta-analysis incorporating both animal and clinical studies (*n* = 8) demonstrated significant variations between supratentorial and infratentorial ICP measurement, with gradients directly correlating with lesion topography [6]. These findings were corroborated by Petr et al. in their observational study of posterior fossa pathologies (*n* = 15), which identified clinically meaningful transtentorial pressure differentials between the supratentorial and infratentorial compartments [7]. Such gradients may have critical implications for therapeutic decision-making, particularly in space-occupying cerebellar lesions, in which traditional supratentorial ICP monitoring could potentially underestimate true infratentorial pressure.

Motivated by these anatomical insights and the paucity of prospective data, we designed this multicenter study to address three fundamental objectives: first, to quantify the magnitude and temporal profile of supra-infratentorial pressure gradients in cerebellar stroke; second, to evaluate technical feasibility and safety of direct infratentorial monitoring; and third, to determine whether infratentorial ICP parameters offer superior prognostic value compared with conventional supratentorial measurements. Through simulteanous dual-compartment monitoring in patients with acute cerebellar stroke, we aimed to provide empirical evidence to guide the evolving paradigm of location-specific ICP management.

## Methods

### Clinical Protocol Approval

This prospective, multicentric cohort study received ethical approval from the institutional review board at Rostock University Medical Center in Rostock, Germany (no. A2020-0276). All participating institutions (University Hospital Jena, University Hospital Heidelberg) obtained local ethics committee approval prior to patient enrollment. Written informed consent was obtained from all participants or their legally authorized representatives in accordance with the Declaration of Helsinki.

### Study Population and Data Acquisition

We consecutively enrolled patients with acute hemorrhagic or ischemic cerebellar stroke admitted to three tertiary neurovascular centers in Germany between January 2021 and April 2024. Inclusion criteria consisted of the following:

1. Age ≥ 18 years
2. Radiologically confirmed acute cerebellar stroke (hemorrhagic or ischemic)
3. Clinical indication for both of the following:
4. Supratentorial external ventricular drainage (EVD)
5. Suboccipital decompressive surgery

Exclusion criteria included the following:

1. Concurrent extracerebellar stroke involvement
2. Nonsurgical management candidates
3. Incomplete clinical or radiological data sets

### Clinical Protocol and Interventions

**Fig. 1:**
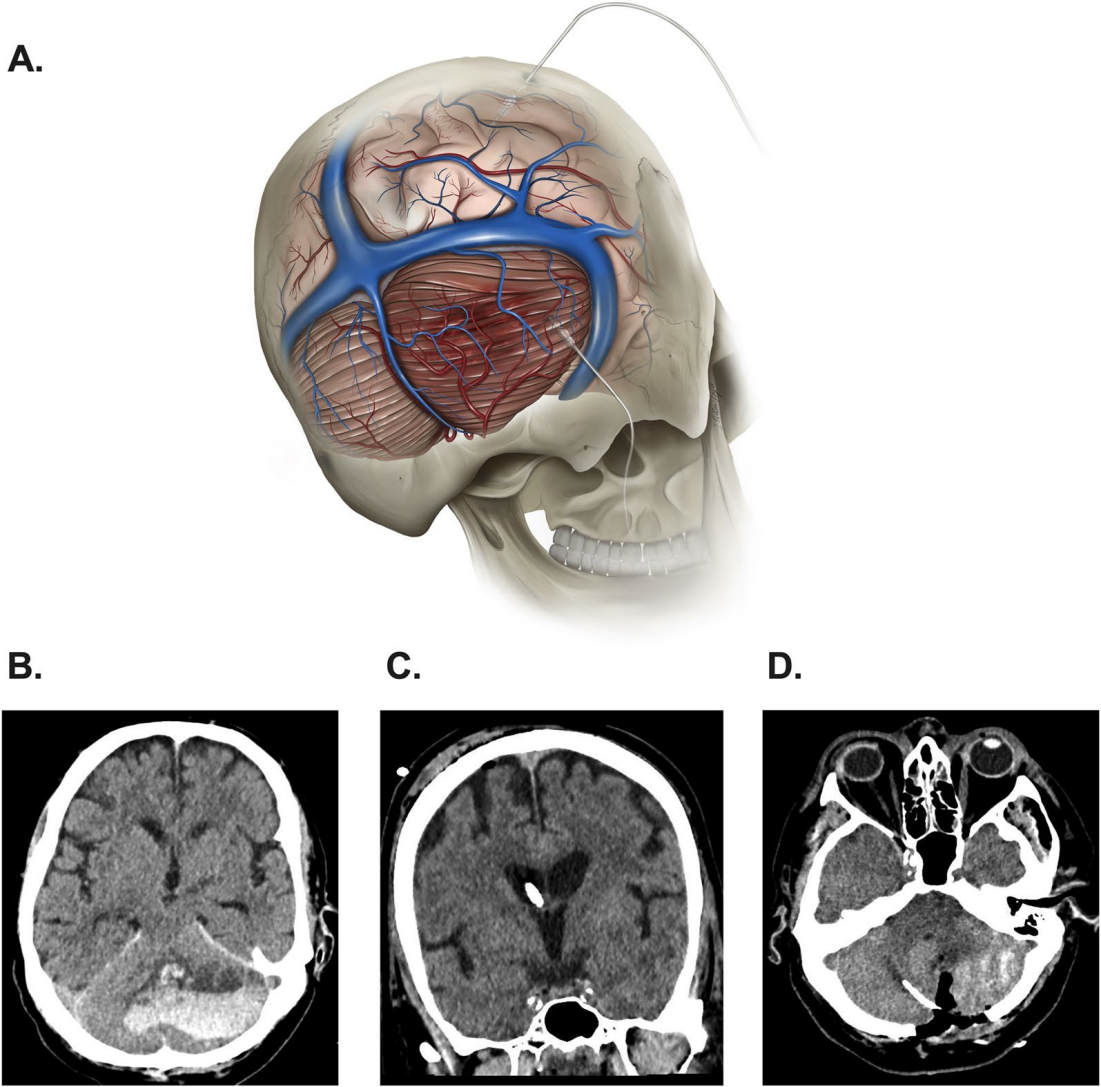
Illustration of simultaneous supratentorial and infratentorial pressure monitoring in a patients with a cerebellar stroke **a**. Axial computed tomography (CT) scan demonstrating a large cerebellar hemorrhage with mass effect and effacement of the fourth centricle **b**. Postoperative axial CT scan showing the supratentorial Spiegelberg external ventricular drainage–intracranial pressure (ICP) probe positioned within the right lateral ventricle **c**. Postoperative axial CT scan confirming the correct placement of the infratentorial intraparenchymal ICP probe within the right cerebellar hemisphere following surgical decompression, craniectomy, and hematoma evacuation (**d**)

All patients were admitted and treated in certified neurological intensive care units. The decision to insert a supratentorial EVD combined with suboccipital decompressive surgery was made based on each institution’s standard clinical protocol independent of the study. Following EVD insertion (or combined EVD-ICP probe from Spiegelberg, Aesculap, Inc., Center Valley, PA) into the lateral ventricle, surgical decompression of the cerebellar hemorrhage or infarction was performed via craniotomy or craniectomy with hematoma or infarct tissue evacuation. Subsequently, an additional ICP 3 PN-sensor from Spiegelberg (Aesculap, Inc.) was inserted in the ipsilateral cerebellum in a medial-to-lateral direction to a depth of approximately 3 cm for infratentorial pressure monitoring. The catheter was tunneled and secured separately to the skin (Fig. 1A–D). One of the benefits of this probe is the automatic zeroing after connection to the monitoring unit; this process is repeated every hour, reducing the risk of a silent zero drift. Following surgical treatment, simultaneous supratentorial and infratentorial pressure monitoring was conducted for up to seven days, with ICP values recorded hourly. The infratentorial monitor was removed at the bedside upon completion of monitoring. The duration of supratentorial EVD was left to the discretion of the attending neurosurgeons.

### Data Collection Protocol

Baseline data consisted of age, sex, diagnosis, Charlson Comorbidity Index, Glasgow Coma Scale (GCS) at admission and discharge, type of surgical treatment, radiological parameter (hemorrhage or infarct volume, intraventricular hemorrhage) and complications. Data were collected using an electronic database (SAP, Germany and COPRA, COPRA System GmbH, Berlin, Germany). Lesion volumes were calculated by the ABC/2 formula.

The functional outcome was assessed six months after stroke via telephone interview or outpatient clinic visit using modified Rankin scale (mRS). Outcomes were determined at hospital discharge and at the six-month follow-up (FU). A favorable outcome was defined as an mRS score of 0–2.

### Primary and Secondary Outcome(s)

The primary objectives of this study were, first, to systematically evaluate pressure differentials between supratentorial and infratentorial compartments through simultaneous ICP monitoring in patients with cerebellar stroke and, second, to determine whether infratentorial ICP values correlate with functional outcomes at FU.

The secondary objectives included the development of a robust prognostic model that could establish clinically significant infratentorial ICP thresholds for outcome prediction and a comprehensive assessment of potential complications associated with the invasive dual-compartment monitoring procedure. The ultimate aim was to advance the understanding of posterior fossa pathophysiology and to improve clinical management strategies for patients with cerebellar stroke.

### Statistical Analyses

Logistic regression and mixed-effects logistic regression models were used to evaluate the predictive information of several markers from the supratentorial and infratentorial pressure measurements as well as nonparametric receiver operating characteristic (ROC) analysis. The targets were in-hospital death on the one hand and favorable outcome at six months at FU (mRS 0–2 vs. mRS 3–6) in in-hospital survivors on the other. The primary markers that were analyzed were mean and median value of supratentorial and infratentorial ICP over the entire period. Furthermore, a linear mixed-effects regression model was used to compare the supratentorial and infratentorial pressures. Data were illustrated by grouped line plots, smooth mean curves, boxplots, conditional density plots, and ROC curves. For binary analysis, unpaired tests (Mann–Whitney *U*-test and *χ*^*2*^ statistics) were used, and a *P* value $\le$ 0.05 was assumed to be statistically significant. Data were analyzed with R version 4.4.1 (R Foundation for statistical Computing, Vienna, Austria) using the packages pROC, Ime4, and lokern.

## Results

### Study Population

The study cohort comprised 35 consecutive patients with radiologically confirmed cerebellar stroke, including 26 (74.3%) hemorrhagic and 9 (25.7%) ischemic events. The mean age was 67.7 ± 14.3 years, with 40% (*n* = 14) of patients being female. The median GCS score on admission was 10 (range 3–14), and mean cerebellar lesion volume was 34.6 ± 16.9 mL. All participants underwent standardized surgical management involving EVD placement combined with either suboccipital craniotomy (57.1%) or craniectomy (42.9%) for hematoma or infarct evacuation, followed by implantation of a cerebellar intraparenchymal pressure probe. The monitoring system demonstrated an excellent safety profile, with only three minor technical complications observed: two probe malfunctions (5.7%) and one probe displacement (2.9%). Complete demographic and clinical characteristics are presented in Table 1.

**Table 1:** Baseline characteristics, complications and outcome of the study population

Variables	n (%), mean $\pm$ SD
Number of patients	35
Age (years), mean $\pm$ SD	67.7 $\pm$ 14.3
Female	14 (40)
Charlson-comorbidity index, median (95% CI)	2 (1–3)
*Pathology*	
Cerebellar hemorrhage	26 (74.3)
Cerebellar infarct	9 (25.7)
*Radiological parameter*	
Mass volume (ml), mean $\pm$ SD	34.6 $\pm 16$.9
*Side of pathology*	
Right	11 (31.4)
Left	14 (40)
Bilateral	10 (28.6)
*Intraventricular hemorrhage*	
External ventricular drain	35 (100)
Right	31 (88.6)
Bilateral	4 (11.4)
GCS at admission, median (95% CI)	10 (3–14)
ICH-score in cerebellar hemorrhage, median (95% CI)	3 (2–5)
*Surgical treatment*	
Craniotomy and hematoma/infarct tissue	20 (57.1)
Craniectomy and hematoma/infarct tissue evacuation	15 (42.9)
*Complications*	
Reoperation due to rebleeding	1 (2.9)
Wound impairment/infection	4 (11.4)
Liqour fistula	5 (14.3)
*Infratentorial probe-associated complications*	
ICP sonde dislocation	1 (2.9)
ICP sonde defect	2(5.7)
Favorable outcome (mRS 0–2) at discharge	8 (22.9)
Mortality at discharge	9 (25.7)
Favorable outcome (mRS 0–2) at 6 months FU	7/32 (21.9)
Mortality at 6 months FU	13/32 (37.1)

N, number; SAH, subarachnoid hemorrhage; SD, standard deviation; GCS, Glasgow Coma Scale; ICH, intracerebral hemorrhage; CI, confidence intervall; EVD, external ventricular drain; ICP, intracranial pressure; mRS, modified Rankin Scale

### Primary Outcome

**Fig. 2:**
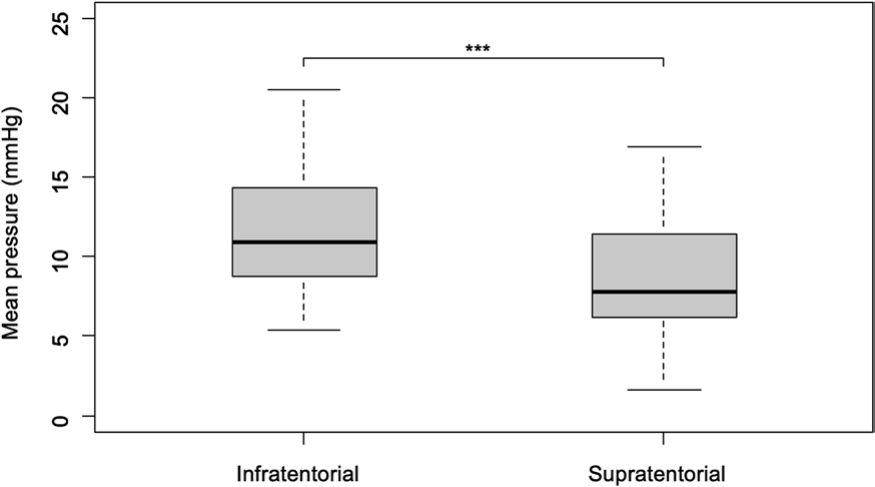
Comparative analysis of supratentorial versus infratentorial intracranial pressure (ICP). Boxplot comparing the mean ICP between the infratentorial and supratentorial compartments across all patients (*n* = 35). The mean infratentorial pressure was significantly higher than the mean supratentorial ICP with a mean difference of 3.2 mm Hg (95% confidence interval [CI] 2.0–4.2, *P* < 0.001). The box represents the interquartile range (IQR), the horizontal line within the box denotes the median, and the whiskers extend to 1.5 IQR

Continuous ICP monitoring yielded 3015 h of supratentorial and 3022 h of infratentorial recordings (mean 86.1 and 86.3 h per patient, respectively). Comparative analysis revealed significantly higher mean ICP values in the infratentorial compartment (11.9 mm Hg, 95% confidence interval [CI] 10.5–13.3) compared with the supratentorial compartment (8.8 mm Hg, 95% CI 7.4–10.1, *P* < 0.001) (Fig. 2). At discharge, 51.4% of patients (*n* = 18) had an unfavorable functional status (mRS 3–6), and the in-hospital mortality was 25% (*n* = 9). Six-month FU data, available for 91.4% of the initial cohort (*n* = 32), demonstrated stable outcome proportions without significant change from discharge status (Table 1).

**Fig. 3:**
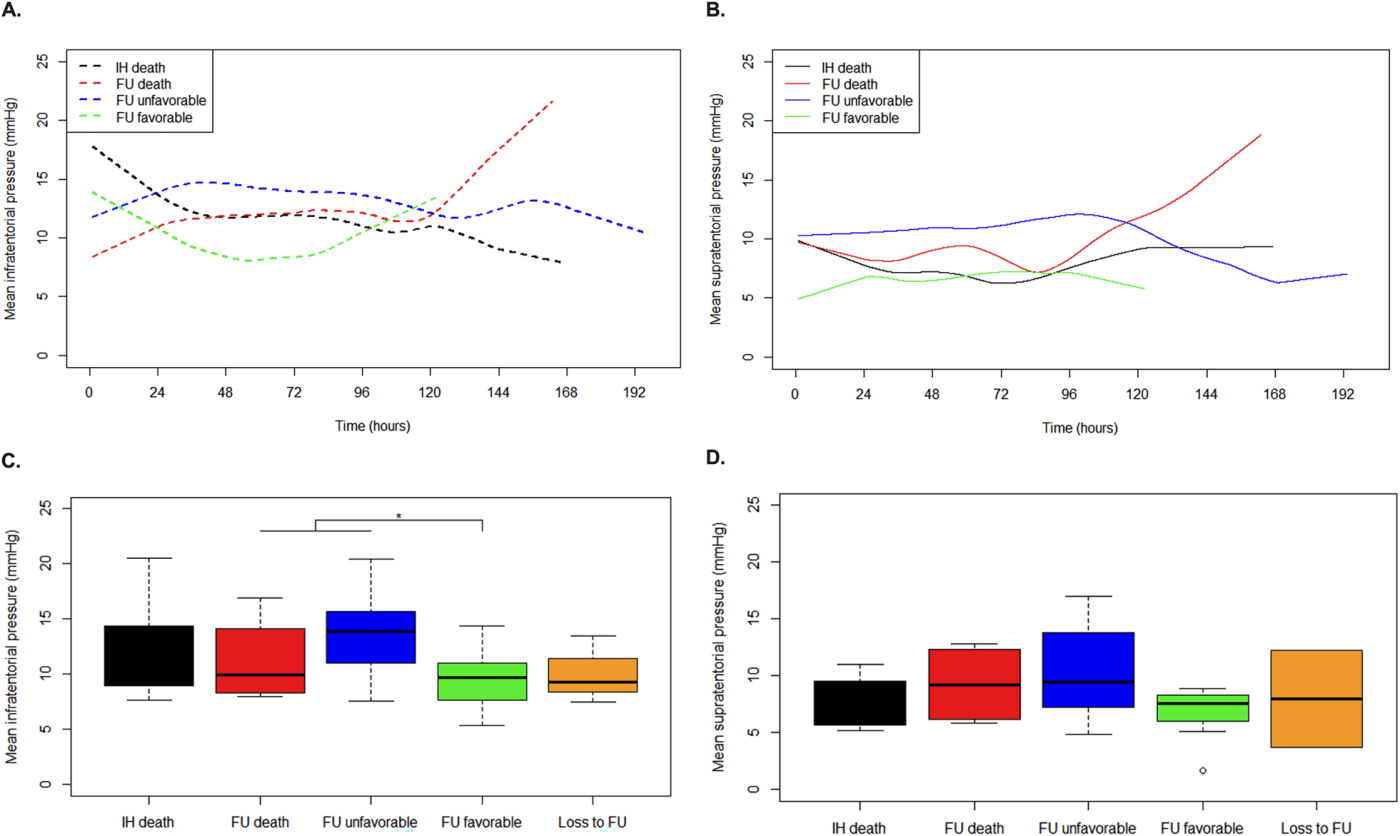
Association of intracranial pressure (ICP) with functional outcome. Smooth mean curve illustrating the temporal profile of **a** Infratentorial and **b** Supratentorial ICP over the monitoring period, stratified by outcome group. In-hospital (IH) death, death at six-month follow-up (FU), unfavorable outcome (modified Rankin scale [mRS] 3–6) at FU, and favorable outcome (mRS 0–2) at FU. Boxplot comparing the mean **c** Infratentorial and **d** Supratentorial ICP values over the entire monitoring period, stratified by outcome group. Patients with unfavorable outcomes had significantly higher mean infratentorial ICP (13.1 mm Hg vs. 9.5 mm Hg, *P* = 0.042), whereas no significant difference was observed in supratentorial ICP (10.5 mm Hg vs. 6.7 mm Hg, *P* = 0.089)

Notably, patients with unfavorable outcomes exhibited significantly higher mean infratentorial ICP values (13.1 ± 3.8 mm Hg, 95% CI 11.1–15.1) compared with those with favorable outcomes (9.5 ± 1.2 mm Hg, 95% CI 6.8–12.1, *P* = 0.042) (Fig. 3, A and C; Supplementary Table 1). In contrast, supratentorial ICP measurements showed no significant association with outcome (10.5 ± 4.0 mm Hg vs. 6.7 ± 2.6 mm Hg; *P* = 0.089) (Fig. 3, B and D; Supplementary Table 2). Comprehensive analyses of outcome predictors are detailed in Table 2.

**Table 2:** Predictors for unfavorable outcome at 6 months follow-up considering in-hospital survived patients

	Unfavorable outcome (%)	Favorable outcome (%)	*p*-value	OR (95% CI)
N	16	7		
Age (years), mean $\pm$ SD	68 $\pm$ 11.2	59.4 $\pm$ 11.8	0.110	NA
Female	6 (37.5)	2 (28.6)	1.0	1.5 (0.2–10.3)
Charlson-comorbidity index, median (95% CI)	2 (1–3)	2 (1–3)	0.884	NA
*Pathology*				
Cerebellar hemorrhage	11 (68.8)	4 (57.1)	0.657	1.7 (0.3–10.3)
Cerebellar infarct	5 (31.2)	3 (42.9)	0.657	0.6 (0.1–3.8)
*Radiological parameter*				
Mass volume (ml), mean$\pm$ SD	38.1 $\pm$ 18.4	30.2 $\pm$ 14.0	0.324	NA
Side of pathology	13 (56.3)	5 (14.3)	1.0	1.7 (0.2–13.7)
Unilateral	3 (18.7)	2 (28.6)	1.0	0.6 (0.1–4.6)
Bilateral	9 (56.3)	3 (42.9)	0.667	1.7 (0.3–10.3)
*Intraventricular hemorrhage*				
GCS at admission, median (95% CI)	10 (3–14)	12 (3–14)	0.398	NA
Intracranial pressure (mmHg)				
Supratentorial, mean $\pm$ SD	10.5 $\pm 4$.0	6.7 $\pm$ 2.6	0.089	NA
Infratentorial, mean $\pm$ SD	13.1 $\pm$ 3.8	9.5 $\pm 1$.2	0.042	NA
*Surgical treatment*				
Craniotomy and hematoma/infarct evacuation	8 (50)	4 (57.1)	1.0	0.8 (0.1–4.5)
Craniectomy and hematoma/infarct tissue evacuation	8 (50)	3 (42.9)	1.0	1.3 (0.2–8.0)

As shown, the mean value of infratentorial and supratentorial intracranial pressure were significant predictors for unfavorable outcome

OR, odds ratio; CI, confidence intervall; n, number; SD, standard deviation; SAH, subarachnoid hemorrhage; GCS, Glasgow Coma Scale

### Secondary Outcome

**Fig. 4:**
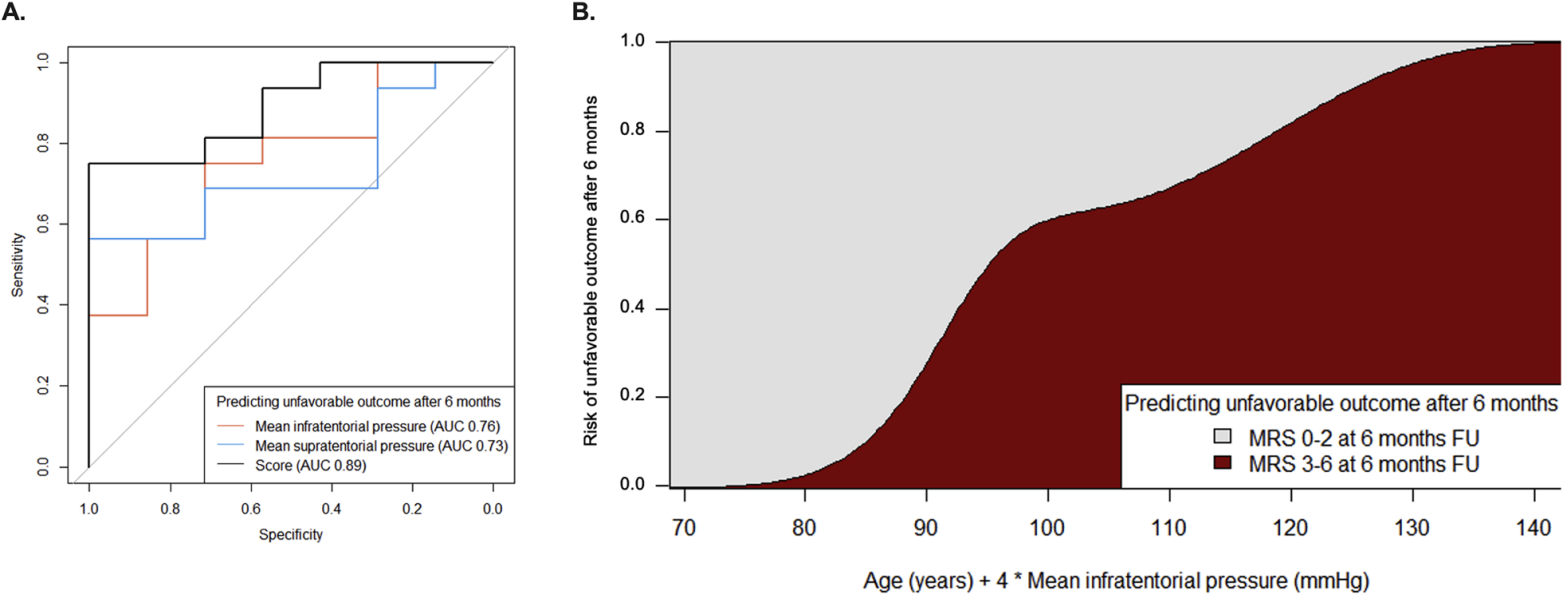
Prognostic performance of the Age/Infratentorial ICP/Cerebellar Stroke (AIICS) score. Receiver operating characteristic curve analysis for the prediction of an unfavorable outcome (modified Rankin scale [mRS] 3–6) at six-month follow-up (**a**). The AIICS score, calculated as age + (four times mean infratentorial pressure [mm Hg]), demonstrated high predictive accuracy (area under the curve [AUC] = 0.89; *P* = 0.045). Conditional density plot visualizing the probability of an unfavorable outcome based on the AIICS score (**b**)

Multivariate logistic regression analysis identified mean infratentorial ICP as the strongest independent predictor of an unfavorable outcome at both discharge (*P* = 0.030, AUC = 0.88) and six-month FU (*P* = 0.055, AUC = 0.89) (Supplementary Fig. 3A; Fig. 4A). Supratentorial ICP values demonstrated weaker predictive capacity (discharge: *P* = 0.079; FU: *P* = 0.070). ICP values from neither compartment were reliable predictors of in-hospital mortality (Supplementary Fig. 4).

### Development of Age/Infratentorial ICP/Cerebellar Stroke Scores

We derived the Age/Infratentorial ICP/Cerebellar Stroke (AIICS) scoring system, calculated as the patient’s age (in years) plus four times the mean infratentorial ICP (mm Hg). This score demonstrated robust predictive value for an unfavorable outcome at discharge (*P* = 0.015) and FU (*P* = 0.045). *t*-Tests confirmed even stronger significance for these associations (*P* < 0.001 and *P* = 0.001, respectively). ROC analysis established 115 as the optimal cutoff value for outcome prediction (*P* = 0.001) (Supplementary Fig. 3B; Fig. 4B). Notably, the model showed no predictive value for in-hospital mortality (*P* = 0.312), and no significant ICP differences were observed between survivors and nonsurvivors.

## Discussion

### Findings

This multicenter prospective cohort study demonstrates that direct infratentorial ICP monitoring in patients with cerebellar stroke is technically feasible and safe, with no significant procedure-related complications observed. Our data reveal consistently higher mean ICP values in the infratentorial compartment compared with simultaneous supratentorial measurements, establishing the existence of clinically relevant transtentorial pressure gradients. Importantly, elevated infratentorial ICP values showed significant association with unfavorable functional outcomes, suggesting that this monitoring modality provides critical pathophysiological insights for managing posterior fossa pathologies.

### Pathophysiological Considerations

Cerebellar stroke represents life-threatening neurological emergencies, with mortality in untreated cases approaching 85% due to malignant edema, brain stem compression, and occlusive hydrocephalus [8]. Although current management paradigms emphasize prompt surgical intervention, our understanding of postioer fossa pressure dynamics remains incomplete [8–10]. The traditional Monro–Kellie doctrine, which conceptualizes the intracranial space as a single closed system maintaining equilibrium between brain tissue, cerebrospinal fluid, and blood volume [11, 12], fails to account for the compartmentalized pressure gradients demontrated in our study corroborated by recent investigations [6, 13–16].

### Clinical Thresholds and the AIICS Score

Current International Brain Trauma Foundation guideline recommend intervention for supratentorial ICP exceeding 22 mm Hg [1], although recent evidence suggests this threshold may require downward adjustment to 18 mm Hg for certain patient subgroups (e.g., older adults, female patients, or patients with a reduced GCS at admission) [17]. The numerous variables influencing ICP limit the generalization of a single threshold. Furthermore, it is questionable whether thresholds derived from supratentorial measurements can be extrapolated to posterior fossa pathologies. To address this limitation, we developed the AIICS prognostic scoring system. This model incorporates patient age, given its established prognostic value for functional outcomes [18, 19], and its association with age-related neuroanatomical changes. Consequently, this model proposes lower ICP treatment thresholds for patients who are older, reflecting their diminished intracranial compliance.

### Clinical Implications and Future Directions

The AIICS score holds significant potential to inform critical decision-making in several high-stakes clinical scenarios. In the neurocritical care unit, a rising AIICS score could provide an objective trigger for escalating therapy, such as initiating advanced medical treatment or proceeding to surgical treatment to mitigate infratentorial hypertension before irreversible damage occurs. Although a limitation of this study was the postoperative timing of ICP monitoring, performing infratentorial monitoring prior to surgery could offer invaluable data for determining the optimal timing of intervention (e.g., surgical decompression); a rapidly escalating infratentorial ICP might be cause for earlier surgery before clinical deterioration becomes apparent. Furthermore, in resource-limited settings or mass-casualty events, the score could serve as a triage tool in the intensive care unit, helping to identify those patients with posterior fossa pathology at greatest risk of rapid decompensation and prioritizing them for higher levels of care. However, it is crucial to acknowledge the current limitations that prevent its immediate standalone use. The AIICS score requires validation in larger, multicenter prospective cohorts to confirm its generalizability. Its performance must also be tested against and integrated with existing multimodal monitoring data (e.g., clinical examination, imaging, and other ICP parameters) rather than replacing them. Ultimately, although not yet a definitive clinical tool, the AIICS score represents a promising step toward quantitative, imaging-derived assessment of posterior fossa compliance that could augment clinical judgment and improve patient outcomes.

### Technical and Safety Considerations

Although concerns persist regarding potential injury to critical structures in the posterior fossa region during probe placement [7, 13, 15], our study adds to the growing evidence supporting the safety of this procedure [6]. Both direct visualization during decompressive surgery and image-guided burr hole placement using the anatomical safe zone described by Vanaclocha et al. (2 cm posterior to the mastoid tip and 2 cm caudal to the transverse sinus) [20] appear to be viable techniques, although further prospectives studies are warranted.

## Limitations

There are several limitations: Firstly, only postoperative supratentorial and infratentorial ICP monitoring data were available (after surgical decompression). Preoperative “base-line” ICP monitoring could have revealed divergent pressure gradients compared with postoperative values, which might be particularly informative to understand the pathophysiology in “non-surgical” patients. Furthermore, the number of patients (*n* = 35) enrolled in this study was not large, which requires extension in a larger study series. Secondly, the hourly frequency of ICP recordings may have missed transient peaks, potently limiting the accurate capture of real-time dynamics in the relationship between supratentorial and infratentorial pressures. Third, the observed pressure difference could theoretically depend on the hydrostatic effects from a higher cerebrospinal fluid column. However, Petr et al. investigated the hydrostatic influence in the dual-ICP trial and found no significant correlation between the transtentorial pressure gradient and the vertical distance between probes [7]. Therefore, although hydrostatics may influence absolute ICP, it is unlikely to explain the significant intercompartmental difference we observed. Fourth, as an observational study, neurointensive management was based solely on the supratentorial ICP values, as no guidelines exist for infratentorial ICP management. Consequently, this study cannot determine how outcomes might be improved if treatments were guided by infratentorial ICP—a critical question for future prospective trials.

## Conclusions

Our findings establish infratentorial ICP monitoring as a safe and clinically informative tool for managing cerebellar stroke. The demonstrated association between elevated infratentorial pressures and poorer outcomes, coupled with the predictive utility of the AIICS score, underscores the need for compartment-specific treatment paradigms. Future randomized controlled trials should evaluate whether therapy guided by infratentorial ICP thresholds can improve functional outcomes in patients with posterior fossa pathologies.

## Supplementary Information

Below is the link to the electronic supplementary material.

Supplementary file1 (PDF 739 KB)

## Data Availability

The data sets used and/or analyzed in current study are available upon reasonable request.
