# Supplementary material for: Infratentorial Pressure Monitoring in Cerebellar Stroke: Feasibility and Prognostic Utility
Source: Neurocrit Care. 2025 Oct 9;44(2):578–86. doi: 10.1007/s12028-025-02391-1 (PMC13053480; doi:10.1007/s12028-025-02391-1)

Supplementary Files

Supplementary Table 1. Infratentorial pressure depending on the outcome of patients.

Supplementary Table 2. Supratentorial pressure depending on the outcome of patients.

Supplementary Figure 1. Infratentorial pressure monitoring during the entire study period divided by the outcome of patients: Green, favorable outcome at 6 months follow-up (FU), Orange, loss of FU; Black, inhospital death; Red, death at FU; Blue, unfavorable outcome at FU.

Supplementary Figure 2. Supratentorial pressure monitoring during the entire study period divided by the outcome of patients as mentioned in *Supplementary Figure 1.*

Supplementary Figure 3. A. Multivariable and ROC-analysis indicated a score with age + 4x mean infratentorial pressure (mmHg) as the most predictive model for unfavorable outcome at discharge ($p=0.015$; AUC 0.88). **B.** The conditional density plot shows the risk of unfavorable outcome at discharge. X-axis is defined as continuous score system and Y-axis as the risk for unfavorable outcome at discharge.

Supplementary Figure 4. A. ROC-analysis for mean infra- and supratentorial ICP taking age into account without any significance ($p>0.05$). **B.** The conditional density plot shows the probability leading to inhospital death when using the score system with age + 4x mean infratentorial pressure (mmHg)

Supplementary Tables

Supplementary Table 1. Infratentorial pressure depending on the outcome of patients

Outcome	Mean ± SD infratentorial pressure (mmHg)	95% CI (mmHg)
IH death	12.2 ± 4.6	7.9 – 16.5
FU death	11.2 ± 4.1	4.7 – 17.7
FU unfavorable	13.1 ± 3.8	11.1 – 15.1
FU favorable	9.5 ± 1.2	6.8 – 12.1
Loss to FU	10.1 ± 3.1	n.a.

SD, standard deviation; CI, confidence interval; IH, inhospital death; FU, follow-up; n.a., not available.

Supplementary Table 2. Supratentorial pressure depending on the outcome of patients

Outcome	Mean ± SD supratentorial pressure (mmHg)	95% CI supratentorial pressure (mmHg)
IH death	7.7 ± 2.3	5.3 – 10.0
FU death	9.3 ± 3.6	3.6 – 14.9
FU unfavorable	10.5 ± 4.0	8.0 – 13.1
FU favorable	6.7 ± 2.6	4.3 – 9.1
Loss to FU	8.0 ± 6.0	n.a.

SD, standard deviation; CI, confidence interval; IH, inhospital death; FU, follow-up; n.a., not available.

Supplementary Figures

Supplementary Figure 1.

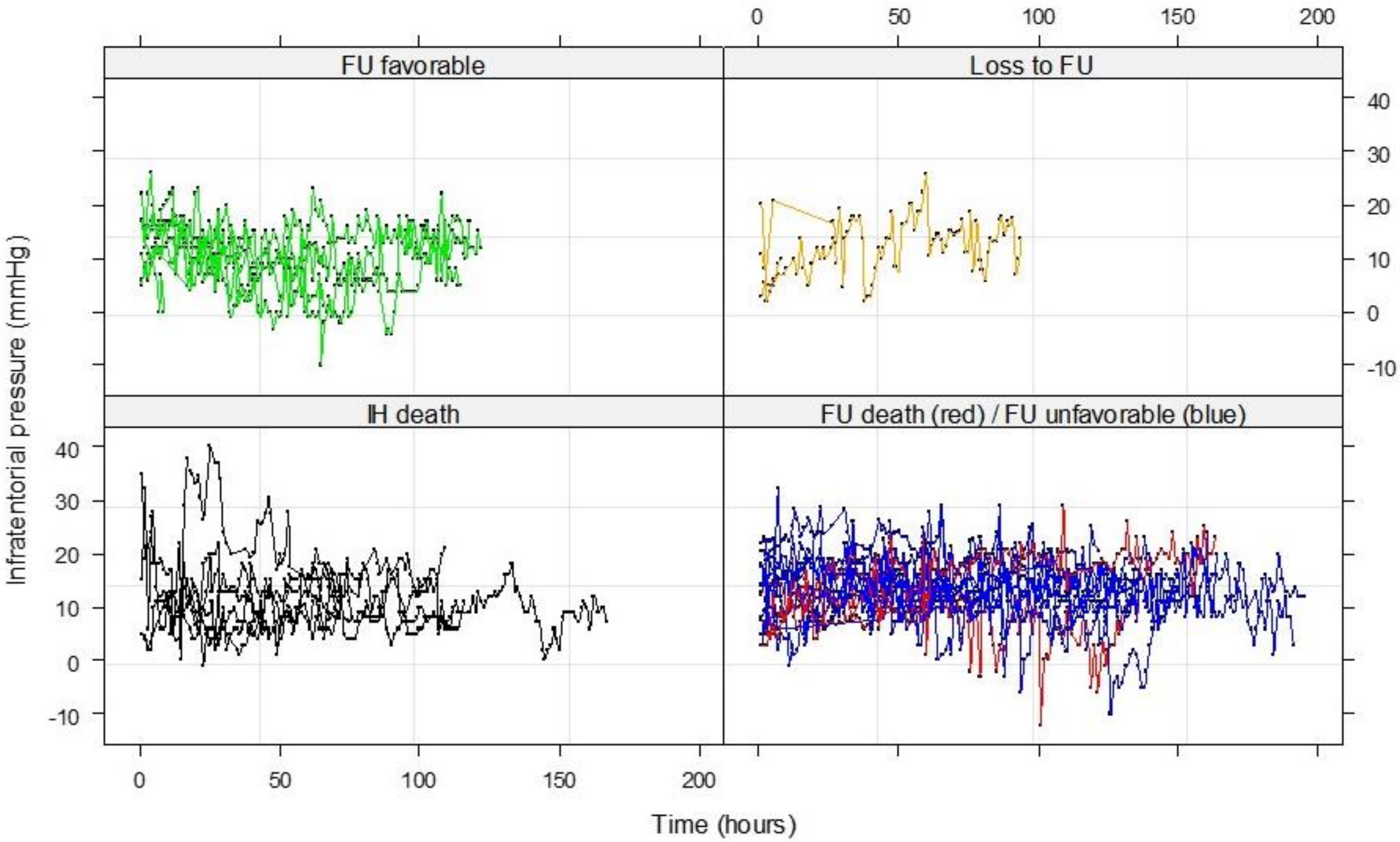

Supplementary Figure 2.

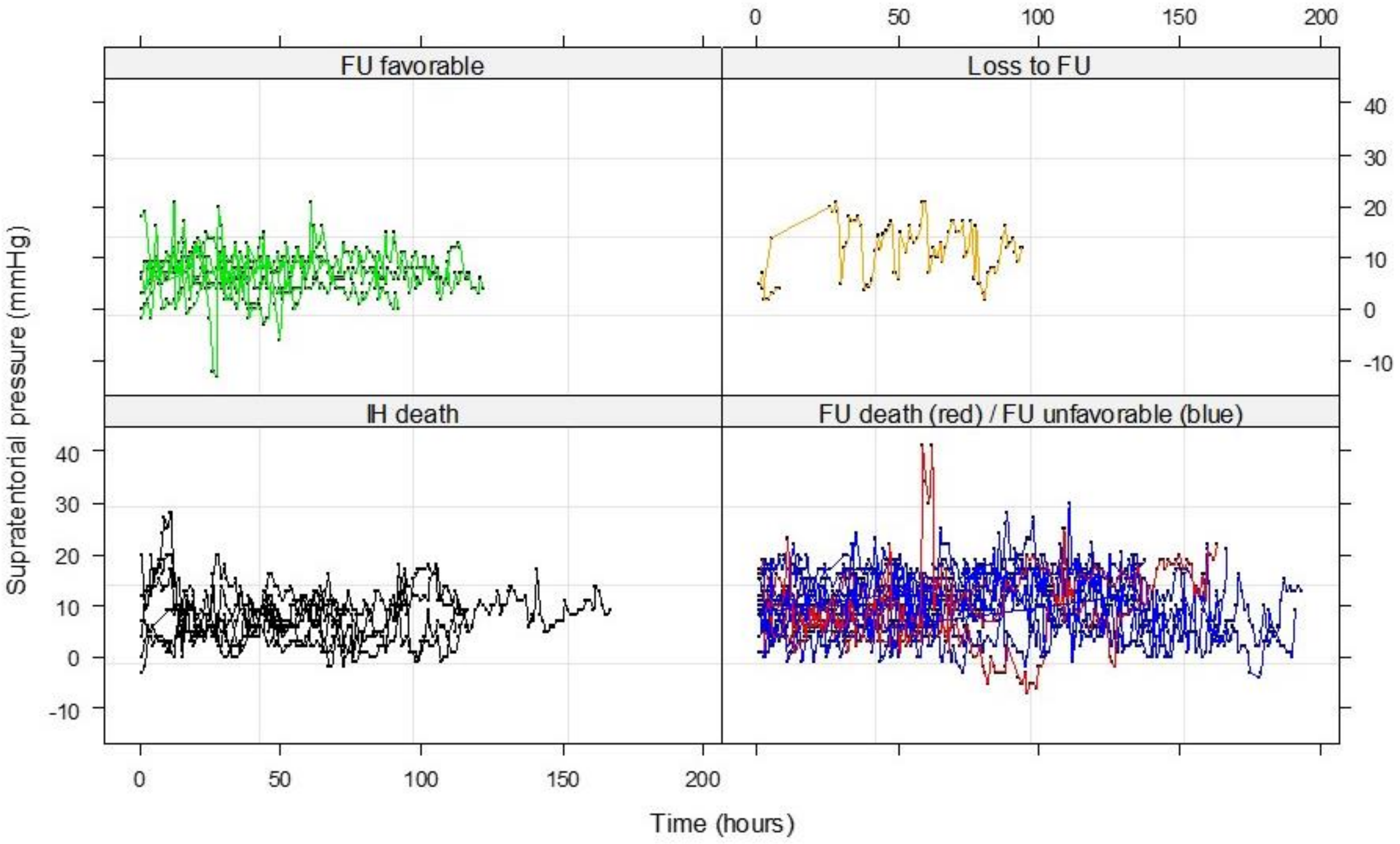

Supplementary Figure 3.

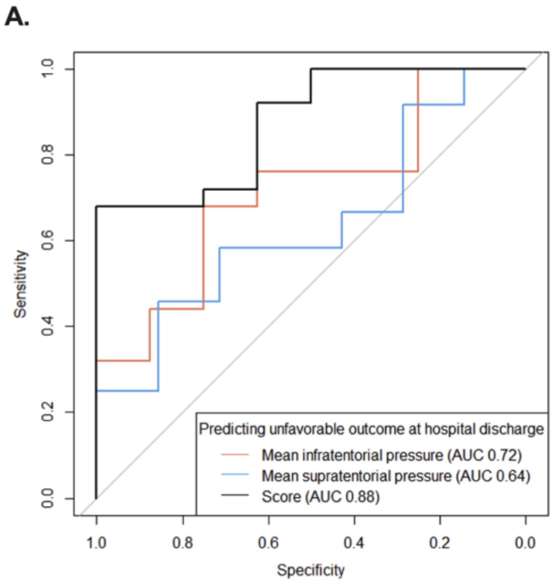

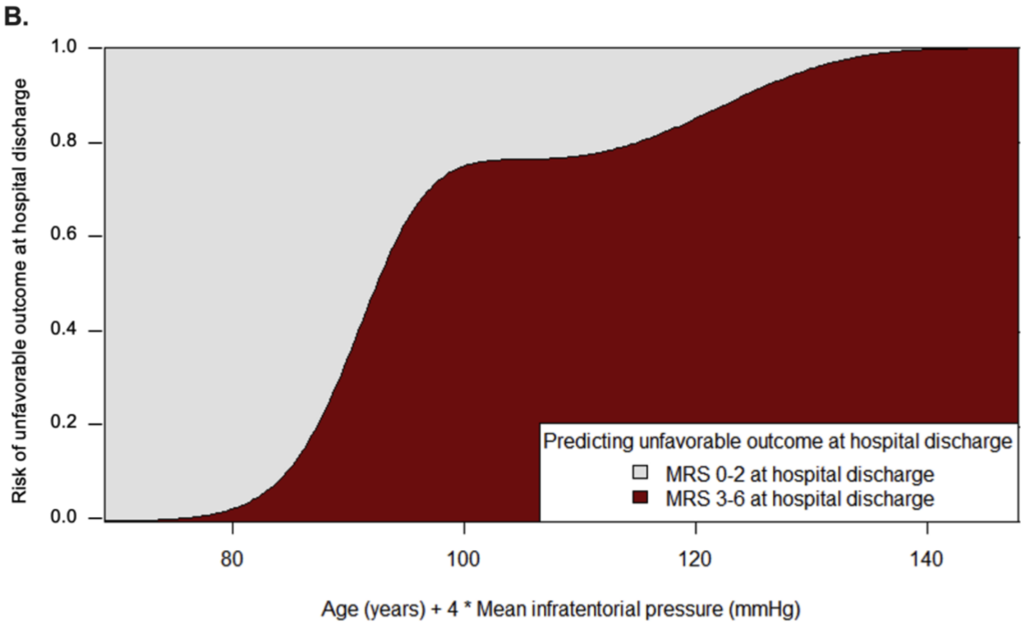

Supplementary Figure 4.

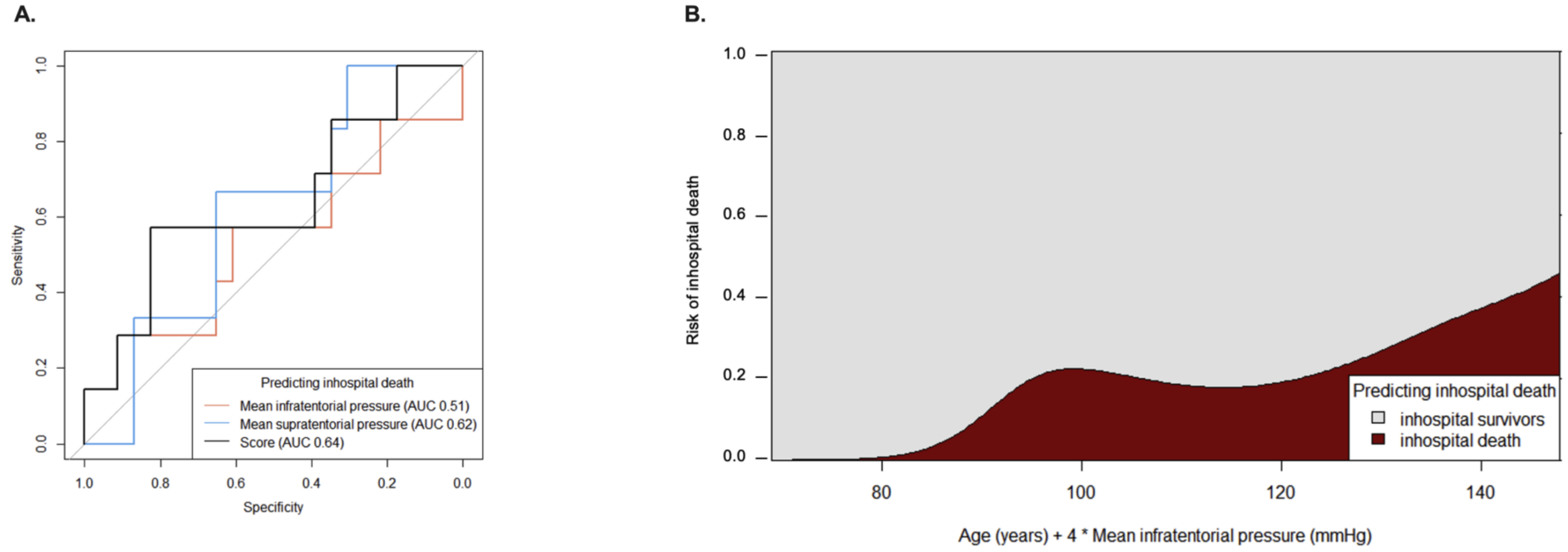

Supplement: Supplementary file 1 — Supplementary file1 (PDF 739 KB) [file 12028_2025_2391_MOESM1_ESM.pdf]
